# Case report: Multiple brain metastases of atrial myxoma: Clinical experience and literature review

**DOI:** 10.3389/fneur.2022.1046441

**Published:** 2023-02-08

**Authors:** Kang Ma, Dawei Zhao, Xuegang Li, Haijun Duan, Chaojun Yan, Shi Wang, Lan Zeng, Kai Xu, Ying Lai, Beike Chen, Ning Mu, Chuanyan Yang, Yulian Quan, Zhengyang Li, Xiaoming Wang, Hua Feng, Fei Li

**Affiliations:** ^1^Department of Neurosurgery and Key Laboratory of Neurotrauma, Southwest Hospital, Third Military Medical University (Army Medical University), Chongqing, China; ^2^Department of Neurosurgery, Chongqing Sanbo Changan Hospital, Chongqing, China; ^3^Department of Cardiosurgery, Southwest Hospital, Third Military Medical University (Army Medical University), Chongqing, China; ^4^Department of Neurology, Affiliated Hospital of North Sichuan Medical College, Nanchong, China

**Keywords:** cardiac myxoma, multiple brain metastasis, gamma knife radiosurgery, temozolomide, cerebellar hemisphere infarction

## Abstract

Myxoma is the most common type of benign cardiac tumor in adults, and it has a strong tendency to embolize or metastasize to distant organs. Patients with multiple brain metastases have rarely been seen in clinics; hence, standard treatment protocols for multimyxoma metastasis in the brain have not been established. We present the case of a 47-year-old female who had convulsions in the right hand and repeated seizures. Computed tomography revealed multiple tumor sites in her brain. Craniotomy was conducted to remove the tumor sites. However, recurrent brain tumors and unexpected cerebral infarctions occurred frequently shortly after the treatment because the cardiac myxoma had not been treated due to the patient's personal concerns. The myxoma was resected by gamma knife radiosurgery, and temozolomide was given prior to cardiac surgery. There has been no evidence of tumor recurrence from the 2 years following the surgery until the present. This case highlights the importance of prioritizing cardiac lesions over cerebral lesions; if a cerebral metastasis has been found, it is likely that the cardiac myxoma is already unstable, with high rates of spread and metastasis. Therefore, it is unwise to treat metastasis sites before the cardiac myxoma. Additionally, the case suggests that gamma knife radiosurgery combined with temozolomide is effective as treatment for multiple myxoma metastasis in the brain. Compared with conventional cerebral surgery, gamma knife radiosurgery is safer, causes less bleeding, and requires a shorter time for recovery.

## 1. Introduction

Myxoma is the most common type of benign cardiac tumor in adults; it occurs in all age groups, particularly between the third and sixth decades of life (1), and it predominantly occurs in women (2). In general, the outcomes are favorable, with a 20-year survival rate of 85%; furthermore, the recurrence rate of myxoma after resection is as low as 5% (2, 3). Although cardiac myxoma is histologically benign, it may be lethal due to its location in the body and ability to metastasize. Cardiac myxomas may lead to cardiac disease in addition to infective, invasive, and malignant processes (1). Myxomas are typically friable or villous, which leads to a higher risk of embolization. The most common site of embolization is the central nervous system (CNS), followed by bone and skin (4–9). Most reports of cerebral involvement have been secondary to embolic occlusion of the intracranial vessels and, less commonly, secondary to bleeding from fusiform aneurysms arising from subendothelial myxoma deposits (10). However, true parenchymal metastases caused by vessel wall transgressions are very rare. There is no standard treatment for patients with brain metastasis of cardiac myxoma due to the low rate of incidence (10, 11). Herein, we present a rare case of multiple intracranial myxoma metastasis in which the timing of the cardiac and cerebral surgeries was abnormal; we first used radiosurgery combined with temozolomide. We also discuss treatments based on a literature review and our experience with this case of multiple cerebral myxoma metastasis.

## 2. Case presentation

**Figure 1:**
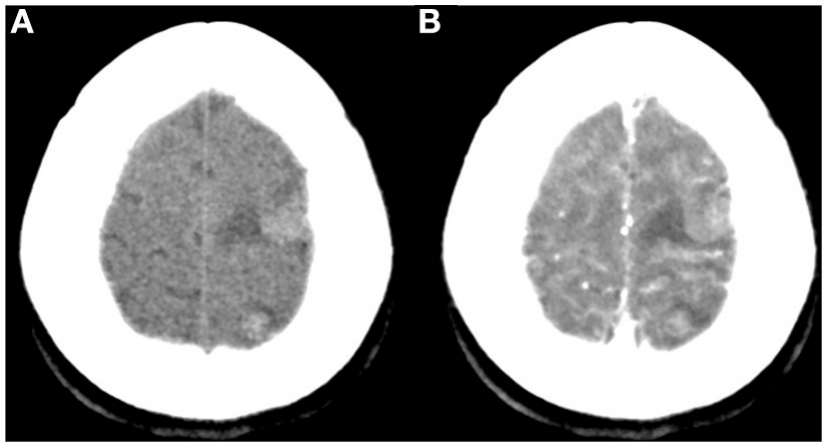
Non-contrast **(A)** and contrast-enhanced CT **(B)** showed multiple rounded lesions with mild inner enhancement in the left frontal and parietal areas.

**Figure 2:**
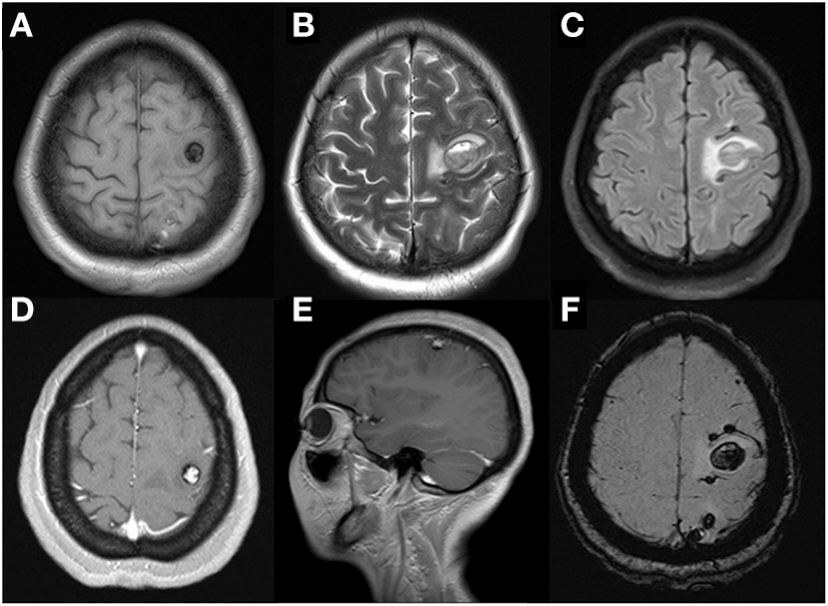
Cerebral MRI examination showed multiple cerebral tumors with recent hemorrhage on axial T1WI **(A)** and T2WI **(B)** and perilesional edema on FLAIR-weighted images **(C)**. Enhanced T1WI after gadolinium showed the largest heterogeneous single-nodular enhancement lesion in frontal lobe on axial **(D)** and sagittal panel **(E)**. That left dispersive presence of multiple focal regions of susceptibility-induced signal loss of variable size on axial SWI **(F)**.

**Figure 3:**
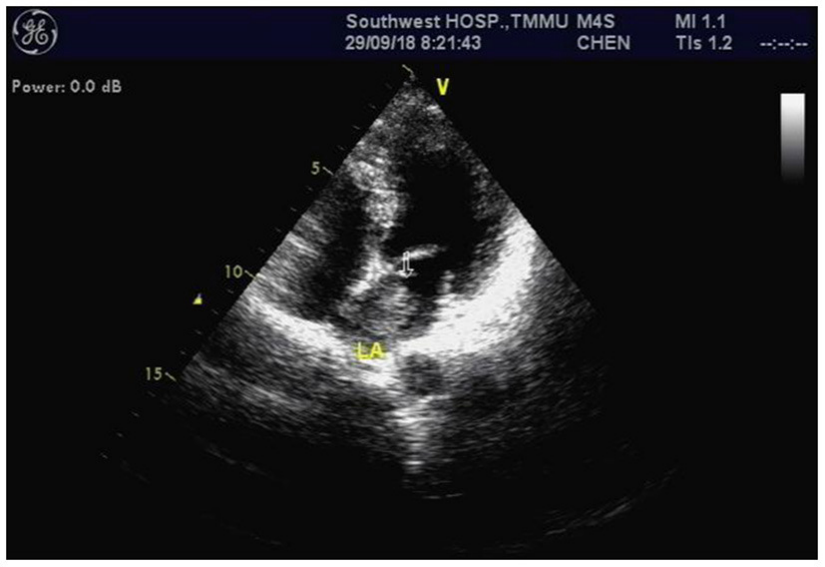
Cardiac echocardiography revealed a left atrium myxoma measuring ~ 27 × 13 millimeters (arrow).

**Figure 4:**
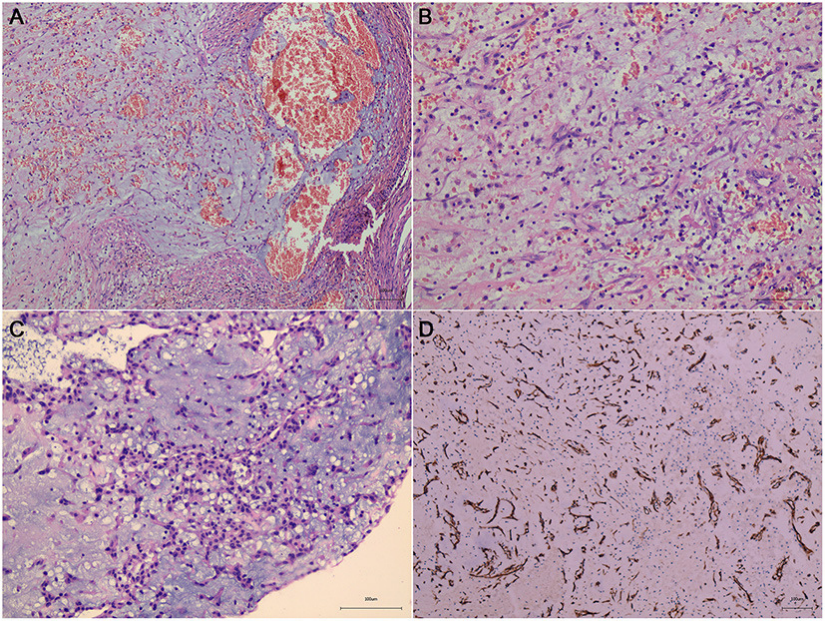
Sections under microscopic examination showed circumscribed fragments of fibrocol-lagenous tissue and some ectatic vascular channels with hemorrhage (**A, B**, metastatic cerebral sites). Under light microscopy, myxomatous component is seen. It is characteristically composed of stellate or fusiform shaped cells surrounded by loose stroma with abundant basophil cells in-filtration (**C**, cardiac myxoma). Immunohistochemical stain revealed positive expression of CD34 **(D)**.

A 47-year-old woman was admitted to our hospital with convulsions in the right hand and repeated generalized seizures occurring up to 6 times a month. Neurological examinations showed that her muscle power was 5 grades below normal in the right limb, accompanied by abnormal sensation. Computed tomography showed multiple high-intensity signals with mild enhancements in the left frontal and parietal lobes (Figures 1A, B). Magnetic resonance imaging showed multiple intracranial tumors with manifestations of bleeding (Figures 2A, B). Enhanced T1WI showed enhanced lesion in frontal lobe (Figures 2D, E). FLAIR (fluid attenuated inversion recovery) and SWI (Susceptibility-weighted imaging) showed peripheral edema around the tumor sites (Figures 2C, F). Overall, the results were consistent with the clinical manifestation of cavernous hemangioma (Figure 3). However, after obtaining cardiac ultrasonography results, the patient was diagnosed with cardiac myxoma with multiple cerebral metastases. We conducted cerebral surgery to remove the tumor sites. The surgery was successful; all four lesions were resected, and the pathological report of the specimens was consistent with pathological features of cardiac myxoma metastases (Figures 4A, B). After the surgery, the patient was given mannitol dehydration and antiepileptic therapy. Although we highly recommended resection of the cardiac myxoma, the patient decided not to remove the myxoma through open-heart surgery for personal reasons.

**Figure 5:**
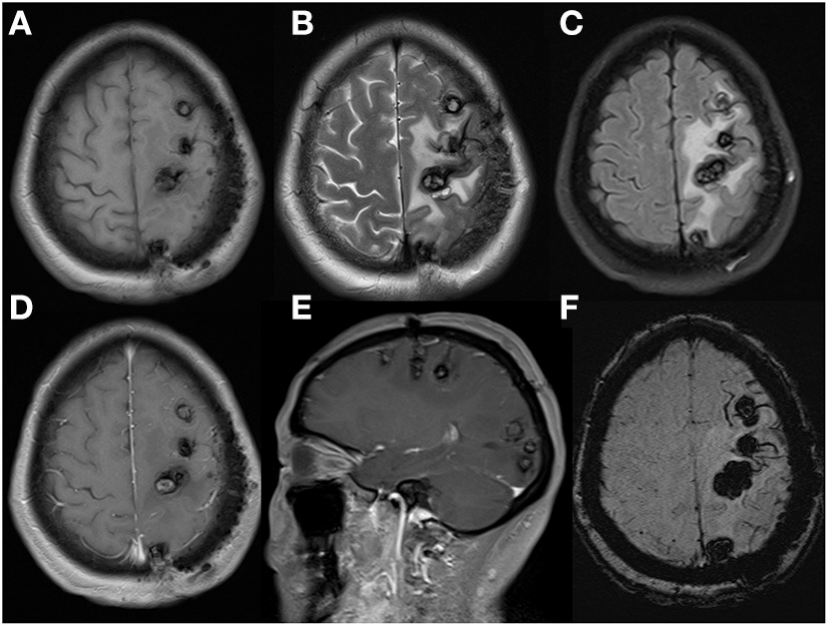
Cerebral MRI re-examination in postoperative 8 months demonstrated cerebral lesions had increased in quantity and size, especially in occipital lobe, with peripheral enlarged edema and mild inner gadolinium enhancement in the largest lesion located in frontal lobe. Axial T1WI **(A)**, axial T2WI **(B)**, FLAIR **(C)**, axial **(D)**, sagittal **(E)**, enhanced T1WI and axial SWI **(F)**.

**Figure 6:**
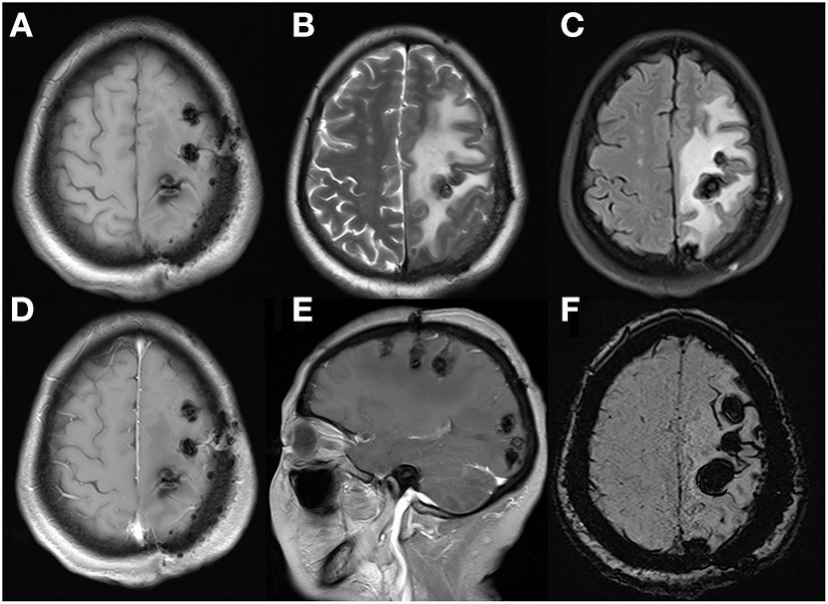
These MRI performed after the second admission revealed serious edema surrounding lesions with mild midline shift, without obvious gadolinium enhancement. Axial T1WI **(A)**, axial T2WI **(B)**, FLAIR **(C)**, axial **(D)**, sagittal **(E)**, and enhanced T1WI and axial SWI **(F)**.

The follow-up MRI results showed that the patient's tumor relapsed only 8 months after the surgery. Moreover, the patient's condition appeared to be worse than it had been prior to treatment, irrespective of the number of lesions and affected lobes. Three new tumor sites in the occipital lobe were found on MRI (Figure 5). At this point, the patient elected to undergo gamma knife radiosurgery for resection of these tumor sites. The post-operative MRI showed that the relapsed tumor sites were entirely removed (Figure 6). Once again, we recommended that the cardiac myxoma be removed immediately through open-heart surgery. However, she declined to undergo cardiac surgery due to personal concerns.

**Figure 7:**
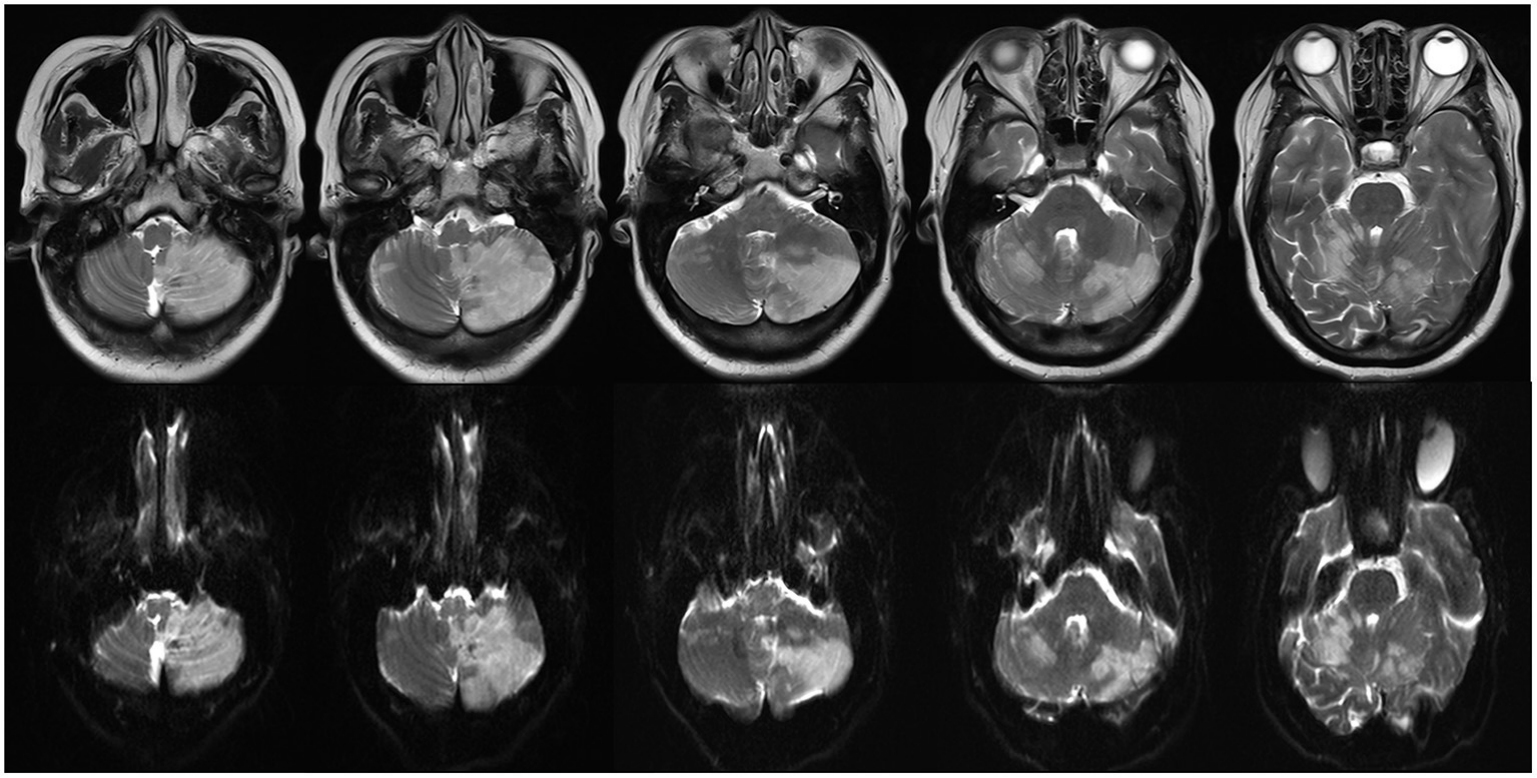
A large bilateral cerebellar hemisphere infarction was identified on multiple panel of T2-weighted **(upper)** and diffuse weighted image **(below)**.

Then, 43 days after radiosurgery, the patient was readmitted to our hospital with symptoms of dizziness, nausea, and vomiting, along with an upper motor type of hemiparesis (power grade 3–5). A cranial MRI revealed severe brain edema without contrast enhancement and mild midline shifting. A diffuse weighted image (DWI) identified massive bilateral cerebellar hemisphere infarction (Figure 7). At this point, the patient finally agreed to our recommendation to undergo cardiac surgery. After considering the possibility of metastatic recurrence, risk of embolisms and aneurysms caused by cardiac myxoma, and evidence that chemotherapy can kill myxomatous cells (12), we suggested that the patient receive our experimental chemotherapy regimen (temozolomide 150 mg/m^2^ for 5 days).

The patient underwent cardiac surgery to remove the atrial myxoma on August 21, 2019. The single gelatinous mucinous tumor found in the left atrium was 40 × 30 × 20 mm in size. The HE report was suggestive of a myxomatous lesion with hemorrhaging (Figure 4C), consistent with cerebral metastatic lesions (Figures 4A, B).

However, some previous cases report that similar multiple brain metastases have been shown in Myxofibrosarcoma patients (13, 14). In this way, differential diagnosis of myxofibrosarcoma must be excluded to conduct a certain diagnosis of myxoma. Myxofibrosarcoma in the heart has been reported to be composed of mitotically active, pleomorphic, and hyperchromatic spindle cells enmeshed within a myxoid stroma and infiltrate the neighboring myocardium. These neoplastic cells are negative for vascular markers and muscular markers (13, 15).

**Figure 8:**
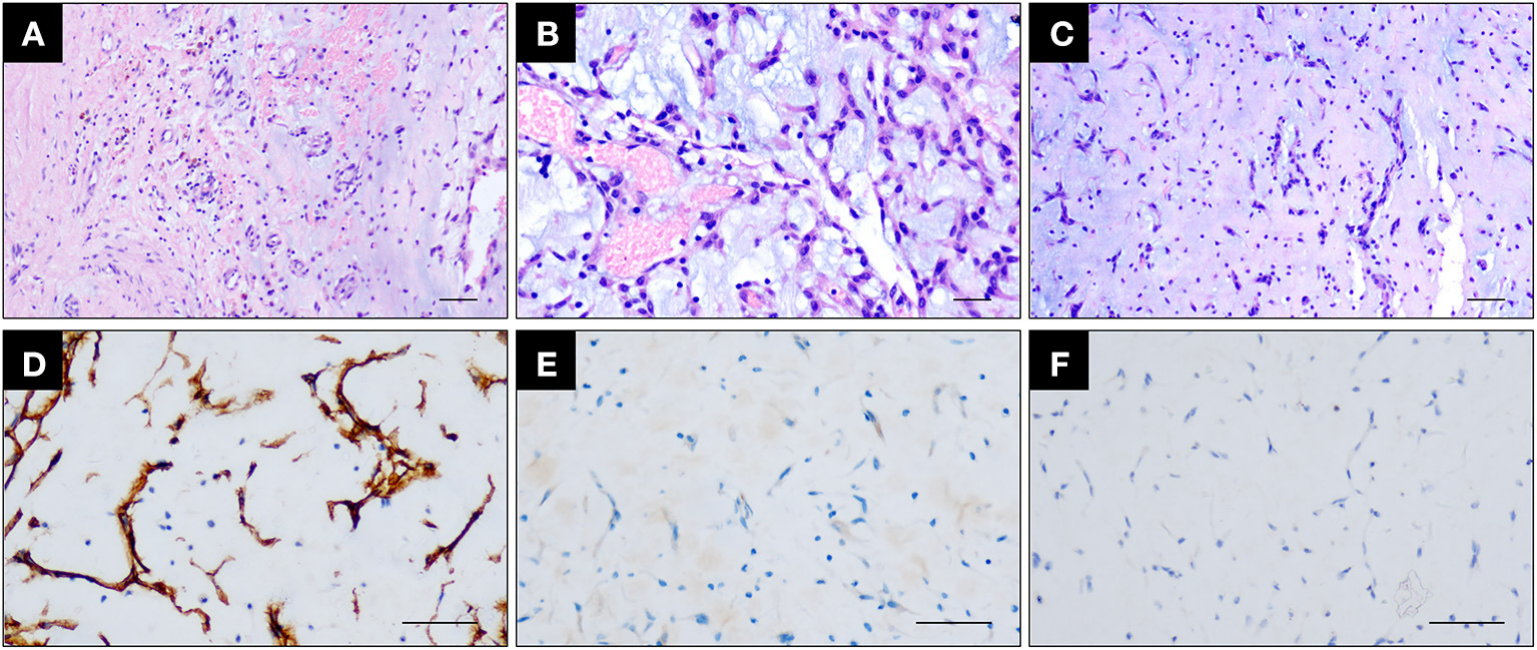
**(A–C)** Hematoxylin and eosin staining showing the cardiac myxoma attached to the atrial wall **(A)** and the enrichment of small plump spindle to ovoid tumor cells with abundant eosinophilic cytoplasm and minimal cytologic atypia **(B)** growing in aggregates and embedded within the myxoid stroma **(C)**. Scale bar = 100 μm. **(D, E)** Immunohistochemical staining showing that myxoma cells express CD34 **(D)** but do not express SMA **(E)**. Scale bar = 100 μm. **(F)** Immunohistochemical staining of Ki67 showing that myxoma cells are with very low proliferation index. Scale bar = 100 μm.

We have conducted hematoxylin and eosin staining and immunohistochemical analysis for differential diagnostic analysis. In this case, the neoplasm is composed of small stellate to plump spindle cells with abundant eosinophilic cytoplasm and the stroma is myxoid (Figures 8A–C), there is no cytologic atypia nor increased mitotic figures, supporting the classical histological features of cardiac myxoma (Figures 8A–C). Immunohistochemical staining showed that myxoma cells in this case highly express vascular marker CD34 (metastatic cerebral site: Figure 4D, cardiac site: Figure 8D), which is not express by myxofibrosarcoma cells. However, these cells do not express the smooth muscle marker SMA or Desmin (Figure 8E). Proliferation index of myxoma cells in this case is very low, i.e., around 1% (Figure 8F). Taken together, we exclude the diagnosis of myxofibrosarcoma as a differential diagnosis.

**Figure 9:**
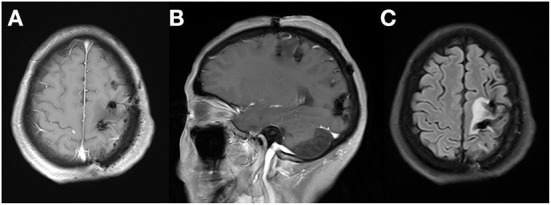
Follow-up MRI revealed no signs of recurrence of tumors and gradually lessening ede-ma surrounding lesions without midline shift and obvious gadolinium enhancement. Axial **(A)**, sagittal **(B)**, and enhanced T1WI, FLAIR **(C)**.

At present, all metastatic sites are well-controlled, and there is no evidence of recurrence (Figure 9). Our patient is clinically well, and the follow-up MRI showed no signs of suspected lesions.

## 3. Discussion

We searched all the studies related to cerebral myxomatous metastases that were published between 1978 and 2022; detailed information from these articles is organized in Supplementary Table 1. The consensus from the literature indicates that cerebral metastasized myxoma can occur at various timepoints, from as early as 2 months (6) to as late as 8 years (8) after cardiac surgery. The biggest treatment differences between our case and reports of previous cases are as follows: (1) the timing of cardiac and cerebral surgeries; (2) the utilization of gamma knife radiosurgery and temozolomide. Although the treatment in our case was explorative, this approach may allow clinicians to better treat patients suffering from multiple cerebral metastases in the future. Considering this, our discussion focuses on the two main differences in treatment between our case and previously reported cases.

### 3.1. The timing of cardiac and cerebral surgeries

The recommended treatment process is to resect cardiac myxoma prior to resecting the other metastasis sites or at the same time (5, 7–9, 16–18). However, due to personal concerns, the patient did not accept cardiac surgery until she was readmitted to our hospital, just 8 months after the cerebral surgery.

Myxomas are typically friable or villous; they typically manifest as a malignant phenotype even though they are predominantly benign (4). The shedding particles of myxoma may cause embolic occlusion of intracranial vessels, weaken arterial walls, and penetrate the damaged endothelium, causing aneurysmal dilation. Additionally, some myxoma particles may form metastatic sites (19). Metastatic lesions can be found in rare cases, even after a cardiac myxoma has been removed entirely because metastatic seeding prior to surgery may give rise to lesions at distant sites (18). These usually present as multiple lesions typically located in the frontoparietal regions (20).

If embolisms, aneurysms or metastatic sites caused by cardiac myxoma are found, the myxoma lesions in the atrium may be considered to be highly unstable, with a very high risk of shedding tumor particles. Therefore, addressing the cardiac myxoma should take priority over other metastatic sites if there are no immediate life-threatening concerns. In addition, the condition of cerebral vessels and organs that are at risk of being metastasized should be closely followed.

### 3.2. Clinical experience of treatment used in this case

Due to the low incidence of multiple cerebral myxoma metastasis, there is no definitive guideline for treatment (5). In clinics, surgery, radiotherapy, and chemotherapy have been used to treat various kinds of tumors (21). Among patients with cerebral metastasis of myxoma, the majority undergo craniotomy to remove the metastatic sites (9, 16, 17). However, in our case, gamma knife radiosurgery was used to remove the recurrent lesions after the patient was readmitted. To our knowledge, this is the first case report of a patient that has undergone gamma-knife radiosurgery for the treatment of multiple metastases of cerebral myxoma. A follow-up MRI revealed that cerebral metastatic myxoma lesions were mostly removed. This outcome suggests that gamma knife radiotherapy is an effective option for treating cerebral metastatic myxoma. In addition, gamma-knife radiotherapy has various advantages over craniotomy, especially for the treatment of benign cerebral tumors (22). However, more studies are still needed to evaluate the safety, efficiency, and risks of utilizing gamma knife radiotherapy to treat cerebral myxomatous metastasis.

We utilized chemotherapy as an adjuvant therapy after the patient was readmitted to the hospital, as there is evidence from multiple cerebral myxomatous aneurysm cases that chemotherapy is effective at killing proliferating myxomatous cells (12, 23, 24). The 2-year follow-up MRI found no recurrent lesions in the patient's brain. The outcome of our case suggests that the combination of temozolomide and radiosurgery can effectively remove and control the recurrence of metastatic myxoma. However, a previous case demonstrated a lack of evidence to support the use of chemotherapy in the treatment of cerebral metastatic myxoma (5). Therefore, further studies are needed to evaluate the efficiency of different kinds of chemotherapy for killing myxomatous cells.

## 4. Conclusions

We recommend that atrial myxoma should be entirely resected as soon as possible following diagnosis. The location of metastatic lesions and the physical condition of the patient should be evaluated to determine the most optimal treatment method. For patients with lesions located in the frontal lobes and parietal lobes, we recommend conducting a craniotomy. In addition, we recommend that patients with lesions in areas that are hard to reach or adjacent to vital functional lobes are treated using radiosurgery; patients in poor physical condition may be benefit from radiosurgery. This case suggests that temozolomide may be an effective chemo-reagent for controlling cerebral myxomatous metastasis. However, further studies and case reports are required to standardize treatment for multiple cerebral metastases of myxoma.

## Data availability statement

The original contributions presented in the study are included in the article/Supplementary material, further inquiries can be directed to the corresponding author.

## Ethics statement

The studies involving human participants were reviewed and approved by Southwest Hospital. The patients/participants provided their written informed consent to participate in this study.

## Author contributions

FL, DZ, and KM: conceptualization. XL and CYan: methodology. SW and LZ: validation. BC and ZL: formal analysis. NM: investigation. CYang and XW: resources. YQ: data curation. KM: writing—original draft preparation. HD, KX, and YL: immunohistochemical staining. FL and KM: writing—review and editing. FL and HF: supervision. FL: project administration and funding acquisition. All authors have read and agreed to the published version of the manuscript.
