# Supplementary material for: Case report: Multiple brain metastases of atrial myxoma: Clinical experience and literature review
Source: Front Neurol. 2023 Feb 8;13:1046441. doi: 10.3389/fneur.2022.1046441 (PMC9944787; doi:10.3389/fneur.2022.1046441)
Supplement: Supplementary file 1 [file Data_Sheet_1.PDF]

Table 1 Intracranial metastases of cardiac myxomas reported in literatures

Authors	Year	Country	Type of article	Age (Years)	Gender	Type of brain lesion on radiology	Type of brain lesion on HP	Location of brain lesion	Sites other than brain	Interval: Primary resection to metastasis	Surgery for brain lesions	Radiotherapy	Chemotherapy	Outcome follow-up
Desousa *et al*[1], Rankin and Desousa[2] and Seo *et al*[3]	1978, 1978, 1980	USA	Case report	44	F	**CT**: Mass	Blood sinusoids with myxoid and connective tissue	Left lateral ventricle-choroid plexus	Multiple bone (both humeri, pelvis, skull, proximal femora, left scapula, left tibia)	8 yrs	Yes	No	No	Survived 6 more years
Budzilovich *et al*[4]	1979		Case report	52	F	**CT**: Mass	Myxoid invasion of neural tissue	Cerebrum (Parietal lobe), dura, cerebellum	No	Primary not resection (postmortem)	Yes	No	No	Dead on arrival (not relevant)
Morimoto *et al*[5]	1986	Japan	Case report	44	M	**CT**: Enhancing low density mass	Clusters of spindle and stellate cells	Cerebrum	Skin	1 yrs	Yes	No	No	
Bazin *et al*[6]	1987		Case report	56	F	**CT**: Multiple high density enhancing	Myxomatous features	Cerebrum, cerebellum	No	4 yrs	Yes	No	No	NK
Kadota *et al*[7]	1987	Japan	Case report	44	M	**CT:** Infarct/ring-enhancing mass	Myxomatous tissue	Cerebrum (left fronto-parietal region)	Skin	1 yrs	Yes	No	No	NK
Ng and Poon[8]	1990	Hong Kong	Case report	55	M	**CT**: Enhancing high density mass	myxoma tissues admixed with hemorrhages, hemosiderin deposits, and fibrosis	Cerebrum (Left occipital region)	No	1 yr	Yes	No	No	moderately disabled
Todo *et al*[9]	1992	Japan	Case report	32	F	**CT:** heterogeneous density and partly cystic mass	sarcoma with a myxomatous change	Cerebrum (right temporal lobe)	Skin	9 months	Yes	Yes (total 60 Gy)	No	Dead
Wada *et al*[10], Kanda *et al*[11]	1993, 1994	Japan	Case report	70	M	**CT**: High density lesions	Benign myxoma	Cerebrum (left and right occipital region)	No	simultaneous	Yes	No	No	Alive
Samaratunga *et al*[12]	1994	Australia	Case report	60	F	**CT**: Enhancing subcortical lesion	Epithelioid haemangioendothelioma with myxoid features	Cerebrum (anterior part of the parietal lobe)	No	Primary not resection	Yes	No	No	Alive
Hui-xia Cao (曹惠霞)[13]	1994	China	Case report	17	F	**CT:** High density lesions		Cerebrum (left parietal lobe)		9 months				
Scarpelli *et al*[14]	1997		Case report	64	M		Metastatic atrial myxoma: glandular variant	Cerebrum (right parieto-occipital)	No	12 yrs	Yes	No	No	NK
Bernet *et al*[15]	1998	Switzerland	Case report	31				Cerebrum (multiple bilateral frontal and occipital lobe)	Soft tissues (scapula and right gastrocnemius muscle), right lung	2 months	No	Total 5000 Gy	Doxorucin and ifosfamide	Alive
Hirudayaraj *et al*[16]	2004	UK	Case report	50	F	**MRI**: enhancing lobulated mass	vascular tumour with fibroblastic areas, some ectatic vascular channels and haemorrhage with very mild cytological pleomorphism without mitoses. (low grade myxosarcoma)	Cerebrum (right parietal region)	No	Primary not resection	Yes	No	No	NK
Altundag *et al*[17]	2005	Turkey	Case report	41	F	**MRI**: multiple cystic lesions **CT**: multiple hemorrhagic lesions	benign myxoma consistent with emboli	Both cerebrum and cerebellum	No	17 months	Yes	30 Gy	No	Alive (follow-up for 4 years)
Rodrigues *et al*[18]	2006	UK	Case report	65	F	**CT**: High density lesions	Metastatic atrial myxoma	Cerebrum (right parietal lobe, both occipital lobes)	No	1 yrs 6 yrs	Yes	No	No	Alive
Moiyadi et al[19]	2007	India	Case report	35	M	**MRI**: heterogeneous intensity, partly solid and cystic multiple lesions	glandular variant of atrial myxoma	Cerebrum (right fronto-parietal, left temporal and occipital lobes)	No	Primary not resection	Yes	25 Gy	No	Alive
Suzuki et al[20]	2008	Japan	Case report	68	M	**CT**: multiple high-density areas			No	6 months	Yes	40.8 Gy whole-brain radiation	No	Alive
Kim et al	2011	Korea	Case report	49	F	**MRI**: multiple hemorrhagic and enhancing lesions with perilesional edema and adjacent superficial siderosis in bilateral cerebral hemispheres		Cerebrum (bilateral cerebral hemispheres)	No	1 yrs	No	35 Gy/24 Fractions whole-brain radiation for 3 weeks	No	NK
Lee et al[21]	2011	Taiwan	Case	33	F	**Enhanced CT**:	brain tissues infiltrated	Cerebrum	No	1 yrs	Yes	No	No	Dead one

			report			multiple mass-like lesions **MRI/MRA**: multiple intracranial tumors and aneurysms	with neoplastic spindle cells	(left frontoparietal, left occipital and right high parietal regions)						week after admission due to brain stem failure
Qiang Zhang (张强) et al[22]	2011	China	Case report	45	F	**CT**: multiple round density lesions with perilesional edema. **MRI**: high intensity with siderosis around lesions	vascular tissues with myxomatous features	Cerebrum (right temporal lobes) Cerebellum	No	15 months	Yes	No	No	Alive
Badrisyah et al[23]	2012	Malaysia	Case report	15	F	**CT:** multiple enhancing solid-cystic lesions with significant perilesional edema	circumscribed fragments of fibrocollagenous tissue with extensive area of hemorrhage and myxomatous component	Cerebrum (right parietal and occipital lobes)	No	3 yrs	Yes	No	No	Alive
Radoi et al[24]	2012	Romania	Case report	45	M	**MRI**: tumours, with heterogeneous gadolinium enhancement, with intratumoural necrosis and calcification, with perilesional oedema	benign myxoma metastasis	Cerebrum (both parietal and left frontal lobes)	No	16 months	Yes	No	No	Alive
Raza and Kamal[25]	2012	Pakistan	Case report	47	F	**MRI**: multiple hyper-intense lesions with surrounding oedema	haemorrhagic infarct, no myxomatous features	Cerebrum Cerebellum	No	4 months	Yes	No	No	Alive
Kierdaszuk et al[26]	2014	Poland	Case report	41	F	**CT**: heterogeneous haemorrhagic lesions surrounded by edema **MRI**: multiple lesions surrounded by edema and contained hemosiderin deposits	hemorrhagic areas with calcifications	Cerebrum (right frontal and parietal lobe, left parietal and occipital lobe)	No	Primary not resection -6 months	Yes	No	No	Alive
Brinjikji et al[27]	2015	USA	Case Series	41	F	**CT**: intraparenchymal hemorrhage with surrounding Edema **MRI**: hemorrhage with surrounding edema and mass effect and a thin enhancing rim **DSA**: small fusiform oncotic aneurysm at a distal left middle cerebral artery branch	Stellate and polygonal tumor cells surrounded by loose stroma with myxoid appearance	Cerebrum (right frontal lobe)	NR	NR	Yes	NR	NR	NK
				34	F	**pre- and post-contrast T1**: two enhancing lesions **Gradient echo**: hypointensity with 'blooming' in the two lesions consistent with blood products **FLAIR**: moderate edema signal surrounding the left posterior temporal lesion **DSA**: two oncotic fusiform aneurysms	NR	Cerebrum (left temporal and posterior left occipital lobes)	NR	2 yrs	NR	NR	NR	NK
Cote et al[28]	2015	Canada	Case report	46	F	**CT:** hyperdense lesions, surrounded by vasogenic oedema, interpreted as hemorrhagic **MRI**: hypointense	metastatic myxomatous disease	Cerebrum (right central sulcus, right inferior frontal gyrus, left precentral gyrus)	No	2 yrs	Yes	No	No	Alive

						lesions								
Castano-Leon et al[29]	2016	Spain	Case report	40	F	**MRI**: four heterogeneous hemorrhagic supratentorial lesions with contrast enhancement and no mass effect	intraparenchymal myxoma	Cerebrum (left pre-rolandic frontal gyrus, left superior parietal lobe, left occipital Lobe) Cerebellum Brain stem	No	Primary not resection	Yes	Holocranial fraction radiotherapy (5 × 150 cGy) receiving a total dose of 35 Gy	No	Alive
Rose et al[30]	2016	UK	Case report	44	M	**CT/MRI**: multiple lesions with mixed irregularities and distortion of the fourth ventricle		Cerebrum Cerebellum	No	5 months	No	30 Gy in 15 fractions whole brain radiotherapy	steroids—Dexamethasone 4mg twice a day	Dead
				52	F	**CT/MRI**: multiple lesions		Cerebrum Cerebellum	No	Primary not resection	No	No	ifosfamide and doxorubicin	NK
Tao Song (宋涛) et al[31]	2018	China	Case report	60	F	**MRI**: heterogeneous signal lesions	Stellate and spindle tumor cells with extensive area of hemorrhage and myxomatous component	Cerebrum (left frontal lobe)	No	Primary not resection	Yes	No	No	Alive
Our case	2020	China	Case report	47	F			Cerebrum (left frontal, parietal, occipital lobes)	No	Primary not resection	Yes	Gamma knife	temozolomide 150 mg/m2 for 5 days	Alive

HP, histopathology; NA, not applicable; NR, not reported; NK, not known; NED – No evidence of disease; DOD – Dead of disease; AWD – Alive with disease.

Reference

[1] DESOUSA A L, MULLER J, CAMPBELL R, et al. Atrial myxoma: a review of the neurological complications, metastases, and recurrences [J]. Journal of neurology, neurosurgery, and psychiatry, 1978, 41(12): 1119-24.

[2] RANKIN L I, DESOUSA A L. Metastatic atrial myxoma presenting as intracranial mass [J]. Chest, 1978, 74(4): 451-2.

[3] SEO I S, WARNER T F, COLYER R A, et al. Metastasizing atrial myxoma [J]. American Journal of Surgical Pathology, 1980, 4(4): 391-9.

[4] BUDZILOVICH G, ALEKSIC S, GRECO A, et al. Malignant cardiac myxoma with cerebral metastases [J]. Surg Neurol, 1979, 11(6): 461-9.

[5] MORIMOTO K, FUJITA T, WAKAYAMA A, et al. Cardiac myxoma metastatic to the brain [J]. Nō to shinkei = Brain and nerve, 1986, 38(9): 865-9.

[6] BAZIN A, PERUZZI P, CHAUFFOUR B, et al. Cardiac myxoma with cerebral metastases [J]. Presse medicale (Paris, France : 1983), 1987, 16(37): 1867.

[7] KADOTA T, IMAKITA S, MITOMO M, et al. Metastatic brain tumor of atrial myxoma [J]. Neuroradiology, 1987, 29(2): 218.

[8] NG H K, POON W S. Cardiac myxoma metastasizing to the brain. Case report [J]. Journal of neurosurgery, 1990, 72(2): 295-8.

[9] TODO T, USUI M, NAGASHIMA K. Cerebral metastasis of malignant cardiac myxoma [J]. Surg Neurol, 1992, 37(5): 374-9.

[10] WADA A, KANDA T, HAYASHI R, et al. Cardiac myxoma metastasized to the brain: potential role of endogenous interleukin-6 [J]. Cardiology, 1993, 83(3): 208-11.

[11] KANDA T, SAKAMAKI T, MURATA K. A cardiac myxoma with interleukin-6 production and cerebral metastasis [J]. International journal of cardiology, 1994, 45(2): 144-6.

[12] SAMARATUNGA H, SEARLE J, COMINOS D, et al. Cerebral metastasis of an atrial myxoma mimicking an epithelioid hemangioendothelioma [J]. The American journal of surgical pathology, 1994, 18(1): 107-11.

[13] 曹惠霞. 左心房粘液瘤脑转移一例 [J]. 中华放射学杂志, 1994, 6):

[14] SCARPELLI M, MONTIRONI R, RICCIUTI R, et al. Cardiac myxoma with glandular elements metastatic to the brain 12 years after the removal of the original tumor [J]. Clinical neuropathology, 1997, 16(4): 190-4.

[15] BERNET F, ., STULZ P M, CARREL T P. Long-term remission after resection, chemotherapy, and irradiation of a metastatic myxoma [J]. Annals of Thoracic Surgery, 1998, 66(5): 1791-2.

[16] HIRUDAYARAJ P, ARYA B, SUVARNA S K, et al. Myxomatous meningeal tumour: a case of "metastatic" cardiac myxoma [J]. International journal of cardiology, 2004, 96(No.3): 471-3.

[17] ALTUNDAG M B, ERTAS G, UCER A R, et al. Brain metastasis of cardiac myxoma: case report and review of the literature [J]. Journal of neuro-oncology, 2005, 75(2): 181-4.

[18] RODRIGUES D, MATTHEWS N, SCOONES D, et al. Recurrent cerebral metastasis from a cardiac myxoma: case report and review of literature [J]. British Journal of Neurosurgery, 2006, 20(5): 318-20.

[19] MOIYADI A V, MOIYADI A A, SAMPATH S, et al. Intracranial metastasis from a glandular variant of atrial myxoma [J]. Acta neurochirurgica, 2007, 149(11): 1157-62.

[20] SUZUKI R, WATANABE T, HIRAYAMA R, et al. Case with cardiac myxoma causing cerebral metastasis after cardiac tumor resection [J]. Kyobu geka The Japanese journal of thoracic surgery, 2008, 61(6): 456-9.

[21] LEE T H, HUANG S C, SU T M, et al. Multiple cerebral aneurysms and brain metastasis from primary cardiac myxosarcoma: a case report and literature review [J]. Chang Gung medical journal, 2011, 34(3): 315-9.

[22] 张强, 胥宝泉, 范祥云, et al. 心房黏液瘤脑转移一例并文献复习 [J]. 中华神经外科杂志, 2011, 27(2): 193-4.

[23] BADRISYAH I, SAIFUL R, RAHMAT H, et al. Brain Metastasis of Atrial Myxoma: Case report [J]. The Medical journal of Malaysia, 2012, 67(6): 613-5.

[24] RADOI M P, STEFANESCU F, ARSENE D. Brain metastases and multiple cerebral aneurysms from cardiac myxoma: case report and review of the literature [J]. Br J Neurosurg, 2012, 26(6): 893-5.

[25] RAZA E, KAMAL A K. Recurrent non-aneurysmal, metastatic intraparenchymal haemorrhages following resection of atrial myxoma – case report and literature review [J]. Bmj Case Reports, 2012, 2012(2012):

[26] KIERDASZUK B, GOGOL P, KOLASA A, et al. Multiple Metastatic Intracranial Lesions Associated with Left Atrial Myxoma [J]. Polish journal of radiology, 2014, 79(2): 262-7.

[27] BRINJIKJI W, MORRIS J M, BROWN R D, et al. Neuroimaging Findings in Cardiac Myxoma Patients: A Single-Center Case Series of 47 Patients [J]. Cerebrovascular diseases (Basel, Switzerland), 2015, 40(1-2): 35-44.

[28] COTE I, SINCLAIR J, WOULFE J, et al. Cerebral Metastasis Presenting after Complete Primary Resection of Atrial Myxoma: Case Report [J]. The Canadian journal of neurological sciences Le journal canadien des sciences neurologiques, 2015, 42(6): 457-60.

[29] CASTANO-LEON A M, HERNANDEZ-LAIN A, MARONAS L, et al. Pathology-confirmed cerebral arterial invasion and recurrent multiple brain metastasis from cardiac myxoma without evidence of disease after surgery and radiotherapy [J]. Clinical neuropathology, 2016, 35(2): 84-8.

[30] ROSE D, PAPA A, TOMAO S, et al. Cerebral Metastases in Patients with Left Atrial Myxoma [J]. Journal of cardiac surgery, 2016, 31(5): 289-93.

[31] 宋涛, 张麒, 姚星星, et al. 左心房黏液瘤的脑转移瘤 1 例 [J]. 中华胸心血管外科杂志, 2018, 34(3): 179-.
